# Comparative Genomics Reveals Accelerated Evolution of Fright Reaction Genes in Ostariophysan Fishes

**DOI:** 10.3389/fgene.2019.01283

**Published:** 2019-12-23

**Authors:** Liandong Yang, Haifeng Jiang, Juan Chen, Yi Lei, Ning Sun, Wenqi Lv, Thomas J. Near, Shunping He

**Affiliations:** ^1^ The Key Laboratory of Aquatic Biodiversity and Conservation of Chinese Academy of Sciences, Institute of Hydrobiology, Chinese Academy of Sciences, Wuhan, China; ^2^ University of Chinese Academy of Sciences, Beijing, China; ^3^ Department of Ecology and Evolutionary Biology and Peabody Museum of Natural History, Yale University, New Haven, CT, United States; ^4^ Institute of Deep Sea Science and Engineering, Chinese Academy of Sciences, Sanya, China; ^5^ Center for Excellence in Animal Evolution and Genetics, Chinese Academy of Sciences, Kunming, China

**Keywords:** ostariophysans, fright reaction, RNA-seq, accelerated evolution, positive selection

## Abstract

The ostariophysian fishes are the most species-rich clade in freshwaters. This diversification has been suggested to be associated with the fright reaction presented in most ostariophysians. However, the genetic forces that underlie fright reaction remains poorly understood. In the present study, through integrating behavioral, physiological, transcriptomic, and evolutionary genomic analyses, we found that the fright reaction has a broad impact on zebrafish at multiple levels, including changes in swimming behaviors, cortisol levels, and gene expression patterns. In total, 1,555 and 1,599 differentially expressed genes were identified in olfactory mucosae and brain of zebrafish, respectively, with a greater number upregulated after the fright reaction. Functional annotation showed that response to stress and signal transduction were strongly represented, which is directly associated with the fright reaction. These differentially expressed genes were shown to be evolved accelerated under the influence of positive selection, indicating that protein-coding evolution has played a major role in fright reaction. We found the basal vomeronasal type 2 receptors (*v2r*) gene, *v2rl1*, displayed significantly decrease expression after fright reaction, which suggests that *v2rs* may be important to detect the alarm substance and induce the fright reaction. Collectively, based on our transcriptome and evolutionary genomics analyses, we suggest that transcriptional plasticity of gene may play an important role in fright reaction in ostariophysian fishes.

## Introduction

As the largest group of fishes in freshwater habitats, Ostariophysi consists of more than 10,000 currently recognized species around the world, which constitutes roughly 75% of all freshwater fish species, 30% of all known bony fish species, and one-sixth of all vertebrate species (Nelson, 2006; Van Der Laan et al., 2014). Fishes from this group include the well-studied “model organism”, the zebrafish (*Danio rerio*), and the famously invasive Asian carps (Cypriniformes). The superorder also includes the enormous Mekong giant catfish (*Pangasianodon gigas*) 2.7 m in length and weighing 300 kg (Siluriformes), well-known electric eel (*Electrophorus electricus*) (Gymnotiformes), and the apex predators piranhas (Characiformes). Considering their ancient origin, enormous evolutionary and ecological diversity, as well as the nearly limitation of all members to freshwater, ostariophysan fishes have attracted a great deal of attention in evolutionary biology and biogeography since the definition of this group (Fink and Fink, 1981; Saitoh et al., 2003; Briggs, 2005; Nakatani et al., 2011; Chen et al., 2013; Arcila et al., 2017; Chakrabarty et al., 2017). Especially, recent analyses have suggested that the common ancestor of ostariophysan fishes entered freshwater about 251 million years ago, which is coincident with, or may be driven by the global decrease in oxygen levels in seawater (Nakatani et al., 2011).

However, with different sets of predator and prey organisms present in the differing habitats between seawater and freshwater, and distinct physical ranges available to them, the ancestor of ostariophysan fishes invading the freshwater habitats should have met with much more living challenges than previous seawater habitats. Interestingly, the ostariophysan fishes have evolved a number of key traits that may help their successful diversification in new freshwater habitats (Nelson, 2006). The first one is that almost all ostariophysan fishes share the Weberian apparatus, which is a specialized modification of the anterior vertebrae and surrounding tissues (Berg, 1912; Rosen and Greenwood, 1970). The Weberian apparatus physically connects the swim bladder to the inner ear, efficiently transfers sound waves between them, and greatly enhances sensitivity to acoustic information (Braun and Grande, 2008). Thus, the ostariophysan fishes specific Weberian apparatus increases their hearing abilities to levels similar to, or even exceeding, those of humans and other mammals (Yan et al., 2000). The second key trait is that most ostariophysan fishes possess a fright reaction, which is elicited by an alarm substance (Schreckstoff) (Frisch, 1938; Pfeiffer, 1963; Smith, 1992). The alarm substance is released from specialized club cells when the skin is injured, which is detected by the sense of smell and causes a fright reaction in nearby members of the same or related species (Frisch, 1938). The fright reaction is important in social communication and predator avoidance, and thus has been suggested to contributed markedly to the biological success of ostariophysan fishes (Pfeiffer, 1977; Helfman et al., 2009). However, the genetic mechanisms of fright reaction in ostariophysan fishes are still unclear. For example, what are the genes responsible for the fright reaction? How many genes change expression levels during the fright reaction?

Besides protein sequence changes, gene expression differences have been suggested to drive many or even most phenotypic innovations (King and Wilson, 1975; Meunier et al., 2013). Comparative gene expression analyses allow the identification of the biological roles underlying the adaptation of organisms to their surrounding environments (Eyres et al., 2016). A large number of studies have stated that the role of gene expression differences in adaptation of phenotypes is controlled by few genes with big effects (Shapiro et al., 2004; Manceau et al., 2011). Nevertheless, the extent to which adaptation of polygenic traits are driven by gene expression differences is not very clear (Fraser et al., 2010; McKinney et al., 2015). Considering that gene expression levels can be adjusted at many steps, and the regulatory networks are complex and modular, changes of gene expression are assumed to be more labile than that of DNA sequence (Huminiecki and Wolfe, 2004). Therefore, changes in gene expression may be particularly important mechanisms by which organisms respond to both short- and long-term changes in specific environmental stresses (Lopez-Maury et al., 2008; Jones et al., 2012).

To investigate the genetic mechanisms that underlie fright reaction in ostariophysian fishes, we employed zebrafish as a model to first examine the effects of alarm substances on behavioural and physiological changes of zebrafish and then used RNA sequencing to quantify the effects of gene expression in the olfactory mucosae and brain. We showed that alarm substances induce dramatically behavioural and physiological reaction, and major gene expression changes in zebrafish after fright reaction. A subset of genes was differentially expressed between control and alarm substances treatments, suggesting differential neurogenomic responses to alarm substances. These genes were enriched for gene ontology (GO) categories tied to stimulus, chemotaxis, and receptor binding. Also, we found significantly accelerated evolution of fright response genes and demonstrated that their rapid evolution is at least partly driven by adaptive evolution.

## Materials and Methods

### Behavior Quantification

To evaluate alarm response, a novel tank diving test was carried out followed the protocol of Cachat (Cachat et al., 2010) with modifications. Briefly, experimentally naive fish was individually transferred and acclimatized for 5–8 min in a transparent tank (15.2 cm height × 22.5 cm bottom × 7.1 cm width) containing 1 L deionized water (DW) at the same temperature as the home tank. To minimize external interferences, 1 ml of DW or alarm substances (AS) released from injured conspecific skin (“skin extract”) (Diaz-Verdugo et al., 2019; Yang et al., 2019) was gently administered to the center of the test tank through a tube about 0.5 cm below the surface of waterline by a syringe. Swimming behavior was recorded using a Video-Track system (ViewPoint Life Sciences, Inc., Montreal, Canada) with a video camera placed 50 cm in front of the tank. Video recording was at 20 frames/s for the first 6 min per fish (n = 20 per group) because zebrafish behavior is most apparent within the first few minutes of novelty exposure (Wong et al., 2010). For analyzing the position and speed of fish swimming, the videos were re-digitized at 20 fps, and the water in tank can be divided into two equal virtual horizontal portions of equal size using the program in the software. Thus, the anxiety-related freezing behavior and erratic movements were quantified by manual registration and computer-aided video-tracking analyses. Freezing was defined as complete immobility at the floor of the tank for at least 1 s. Erratic behavior was defined as a characteristic sharp, rapid change in direction or movement, or repeated darting.

### Cortisol Extraction and Analysis

After AS exposure trial completed, six fishes per group from the AS treated and control zebrafish were used for cortisol extraction. Fish were captured, immediately killed by rapid freezing in liquid nitrogen and then stored at a −80°C freezer.

Whole-body cortisol was extracted using the method described by Cachat et al. (2010). Fish samples were thaw on ice with the head removed using a razor blade. Individual trunks were weighed and placed in 2 ml extraction tube containing 10 zirconia beads. Then the samples were homogenized in 1 ml·(w/v), ice-cold, phosphate-buffered saline (PBS, pH 7.4) for 1 min using a Tissue Cell-destroyer 1000 (NewZongKe, Inc., Wuhan, China). The homogenate in the tube was transferred to labeled tube followed by rinse with an additional 0.5 ml of ice-cold PBS. Cortisol extraction of each sample was conducted with twice of diethyl ether extraction. For each extraction, 5 ml diethyl ether was added to the tube, then vortexed vigorously for 1 min and centrifuged at 4°C for 15 min. The top organic layer containing cortisol was transferred to a glass test tube and evaporated in a fume hood overnight. Before measurement, the lipid extract was reconstituted with 1 ml PBS, vortexed for 30 s and incubated overnight at 4°C.

Cortisol levels were measured in duplicate samples of each extract using a commercially available ELISA kit (Uscn Life Science Inc., Wuhan, China). Accuracy was verified by calculating the recovery from samples spiked with known amounts of cortisol (111.1, 37.04 and 12.35 ng/ml). The recoveries of the spiked samples were 90–104% with relative deviation ≤7.5%. Serial dilutions of the samples were performed to test for linearity, which showed a strong positive correlation between the curves (R^2^ = 0.8924) and the inter- and intra-assay coefficients of variation were 8.1% and 9.7%, respectively. Cortisol metabolites concentrations are expressed in nanograms per gram of fish body weight.

### Generation of RNA-Seq Data

**Figure 1:**
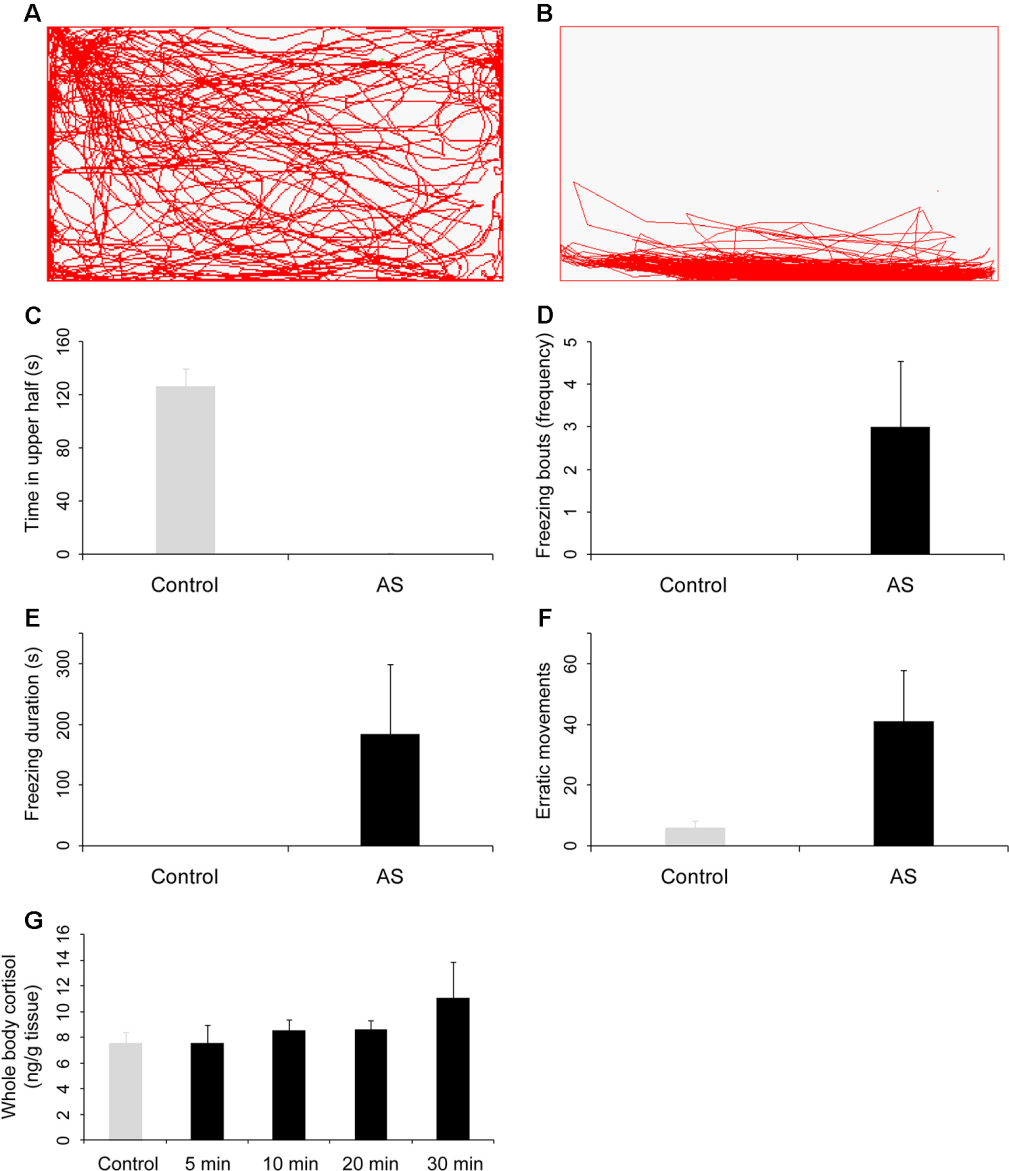
Behavioral and physiological response of zebrafish to the alarm substances (AS). Side-view video of swimming behavior of individual zebrafish before **(A)** and after **(B)** treatment with alarm substances. **(C**–**F)** Graphs of behavioral parameters of fright reaction, including the time in upper half of the test tank **(C)**, the number of freezing bouts **(D)**, the amount of time spent frozen **(E)**, and the number of erratic movements **(F)**. Alarm substances increased the whole body cortisol levels **(G)**. All p values between control and test groups were lower than 0.001.

Procedures for all experiments in this study were approved by the Institutional Animal Care and Use Committee of Institute of Hydrobiology, Chinese Academy of Sciences (Approval ID: Y21304501). Two groups of adult wild-type zebrafish (10 fish per group) from AB background were maintained in the zebrafish facilities for one week to familiarize with the laboratory environment. One group (test) of zebrafish was directly exposed to a size-matched conspecific sample that had just been killed and had skin damage, with the other unexposed group of zebrafish as the control group. When the test group zebrafish showed a severe stress response (**Figure 1D**), they were anesthetized immediately. Olfactory mucosae and brains were dissected out, and frozen in liquid nitrogen quickly. To prevent a possible handling-induced stress response, the time period between capture and killing was short than 30 s. In order to obtain enough RNA for each sample, tissue from three animals was pooled together. Three independent biological replicates for both the control and test groups were prepared.

### Library Construction and High-Throughput Sequencing

Total RNA for each sample was extracted using SV Total RNA Isolation System (Promega). The RNA quality was assessed using agarose gel electrophoresis and RNA integrity was measured using the RNA Nano 6000 Assay Kit of the Agilent Bioanalyzer 2100 system (Agilent Technologies, CA, USA). Sequencing libraries preparation and high throughput sequencing were performed by *Novogene* (Beijing, China) following our previous study (Yang et al., 2015). Briefly, mRNA was purified from total RNA using poly-T oligo-attached magnetic beads and fragmented into short pieces. Subsequently, the first-strand cDNA was synthesized using random hexamer primer and then second-strand cDNA was generated. Finally, the paired-end cDNA library was prepared according to the Illumina’s protocols and sequenced on Illumina HiSeq 4000 platform (150 bp paired-end). The sequencing data have been deposited into the National Center for Biotechnology Information (NCBI) Sequence Read Archive database (Accession No. SRP154651, **Supplementary Table S1**).

### Bioinformatic Analysis of RNA-Seq Data

Raw RNA-seq reads were filtered to remove primer dimers and low-quality bases (Phred quality score lower 20) using the program Trim Galore! (version 0.3.7) (https://www.bioinformatics.babraham.ac.uk/projects/trim_galore/). Only paired-end reads for which either read was longer than 50 bp after trimming were retained for subsequent analysis. High quality paired-end reads from each sample were mapped to the zebrafish reference from Ensembl (release 85) (Cunningham et al., 2015) using Tophat (v2.0.13) (Trapnell et al., 2012; Kim et al., 2013). The assembled transcripts were obtained using Cufflinks and then merged with the annotated reference transcripts using cuffmerge (Trapnell et al., 2012). Cufflinks was run using the GTF file with the parameter “-G” to retrieve expression level for each gene. Differential expression analysis was performed using cuffdiff (Trapnell et al., 2012). Genes with fold change ≥1.5 and adjusted p-value ≤ 0.05 were considered to be differentially expressed. Full lists of differential expression genes can be found in **Supplementary Table S2**.

### Quantitative Real-Time PCR (Qpcr)

To validate the differentially expressed genes identified by RNA-seq, we performed quantitative real-time PCR (qRT-PCR) on a subset of genes that among the significantly DEGs between the AS treated and the control zebrafish. Primers for these genes were designed using the NCBI primer designing tool (http://www.ncbi.nlm.nih.gov/tools/primer). First strand cDNA was synthesized from 500 ng of total RNA samples using M-MLV Reverse Transcriptase (Promega, Madison, WI, USA) and diluted 1:10 as amplification template. qRT-PCR was performed in a 10-μl volume using the LightCycler^®^ 480 SYBR Green I Master on a LightCycler^®^ 480 II Instrument (Roche, Switzerland). Thermocycling conditions were 95°C for 5 min, followed by 45 cycles of 95°C for 20 s and 58°C for 25 s, and a melting curve analysis was performed to confirm the primer specificity after amplification. A list of primers is provided in **Supplementary Table S3** and the elongation factor alpha (*EF1α*) gene was employed as an internal reference. The relative gene expression compared to the control group was determined using the comparative C_T_ method (Livak and Schmittgen, 2001) and the fold change values were the mean of six biological replicates from each group.

### Gene Ontology Analysis

Overrepresentation of the Gene Ontology (GO) terms of the differentially expressed genes were identified using GOrilla (Eden et al., 2009), which allows detecting functional overrepresentation in a candidate data set against a list of background genes. We set the false discovery rate (FDR) of 0.001 as our cutoff value and conducted separately for each tissue.

### Divergence Estimates

To determine the evolutionary rates of differentially expressed gene, we obtained protein-coding gene sequences from zebrafish in Ensembl (release 85), and grass carp (*Ctenopharyngodon idellus*) genome (Wang et al., 2015), and selected the longest transcript of each gene in this analysis. We identified one-to-one orthologs between zebrafish and grass carp using Inparanoid 4.1 (Ostlund et al., 2010) with default parameters and aligned them using PRANK (v.140603) (Loytynoja and Goldman, 2005) at the codon level with the option “-codon”. We employed SWAMP (Version 31-03-14) to filter regions with poor alignment with a cutoff of 4 in a window size of 5, and a minimum length of 75 bp (Harrison et al., 2014). We further excluded the positions having gaps and “N” in the alignments and removed the alignment shorter than 100 bp from analysis. We calculated the number of nonsynonymous substitutions per nonsynonymous site (dN), the number of synonymous substitutions per synonymous site (dS), and their ratio (dN/dS) for each ortholog using codeml program in PAML 4.7 package (free-ratio model, runmode = 2, and model = 1) (Yang, 2007). Orthologs with saturated synonymous substitution values (dS > 2) or N*dN or S*dS < 1 were excluded from subsequent analysis.

The above two-species approaches measure the functional divergence that has occurred between zebrafish and grass carp, which shared a last common ancestor roughly 50 Ma (Wang et al., 2015). As our gene expression datasets were taken from zebrafish, we were also interested in the pattern of substitution that has occurred on the zebrafish lineage alone. In order to do this, we expanded the dN/dS analysis to cover several more taxa: cave fish (*Astyanax mexicanus*), medaka (*Oryzias latipes*), stickleback (*Gasterosteus aculeatus*), and spotted gar (*Lepisosteus oculatus*). We obtained one-to-one orthologs among zebrafish, cave fish, medaka, stickleback, and spotted gar using Biomart and combined them with grass carp to yield 6,846 1:1:1:1:1:1 orthologs among these six species. Orthologs alignment and trimming were conducted in the same manner as the pairwise alignments. We calculated lineage specific evolutionary rates (dN, dS, and dN/dS) on zebrafish using codeml (PAML 4.7 package) with the free-ratio model (runmode = 0 and model = 1) and excluded orthologs with dS > 2 or N*dN or S*dS < 1 from analysis.

### Positive Selection Analysis

To identify evidence of positive selection acting on a subset of sites of the orthologous genes, we used the paired nested site models (M1a vs M2a; M7 vs M8) (Yang, 2000; Yang, 2007) in the codeml program (PAML 4.7 package), which only allow dN/dS ratio to vary among sites in the alignment. The likelihood ratio test (LRT) was employed to compare twice the log likelihood value differences between two nested models (M1a vs. M2a; M7 vs. M8) for each orthologous gene with a chi2 distribution with df = 2. The genes with FDR adjusted p-value <0.05 from one of the two LRT tests were identified as positively selected genes.

As site model only allows dN/dS ratio to vary among sites but not across lineages, we further employed branch-site models (model = 2, NS sites = 2) in codeml program to identify genes under positive selection in zebrafish lineage. Through setting zebrafish as the foreground branch, we compared a neutral model that constrained a class of codons on the zebrafish branch to have ω = 1 (Model A1, fix_omega = 1, omega = 1) with a selection model that allowed this additional class of sites to have ω > 1 (Model A2, fix_omega = 0, omega = 1.5). The LRT test was employed to calculate a p-value from the statistics (twice the log likelihood value differences) and genes with FDR adjusted p-value <0.05 were identified as positively selected genes.

### Synonymous Codon Usage Analysis

Codon usage bias was assessed using two parameters: the frequency of optimal codons (Fop) and the effective number of codons (ENCs). Both parameters for all the differentially expressed genes and unbiased genes identified in this study were calculated using CodonW program (version 1.4.2) (http://codonw.sourceforge.net/). For Fop, higher values suggest stronger synonymous codon usage bias, while for ENCs lower values indicate stronger synonymous codon usage bias (Hambuch and Parsch, 2005).

### Gene Expression Breadth

RNA-seq data sets from a number of different zebrafish tissues and organs, including heart, liver, brain, spleen, kidney, muscle, intestine, gill, blood, gonad, were obtained from NCBI SRA database. Raw reads were filtered and then mapped to zebrafish reference genome as described above. Gene expression levels (FPKM) were estimated by Cufflinks. The specificity index (*τ*) (Yanai et al., 2005) was used as a measure of breadth of gene expression for each gene as follows:

$$\tau =\frac{\sum_{i=1}^{N}\left(1-x_{i}\right)}{N-1},$$

where *N* is the number of tissues, *x_i_* is the FPKM value in the given tissue *i*, normalized by the highest FPKM value of the gene across all analyzed tissues (*N*). *τ* index values range from 0 to 1, with higher *τ* values corresponding to stronger tissue specificity (low expression breadth).

## Results

### Dramatically Behavioral and Physiological Reactions in Zebrafish After Exposed to Alarm Substances

Because of a significant similarity in the basic principles of olfactory representation, the zebrafish has become an important model system for understanding olfaction in vertebrates during recent years (Yoshihara, 2009; Hussain et al., 2013). Thus, we first quantified the behavioral and physiological reactions in zebrafish after exposed to alarm substances (**Figure 1**).

We found that the swimming trajectory of zebrafish was evenly distributed in the rectangular tank when administered with deionized water (**Figure 1A**). However, the zebrafish was completely in the bottom of the tank after exposed to alarm substances, suggesting dramatic fright reaction appeared (**Figure 1B** and **Movies S1**). Specifically, the zebrafish exposed with alarm substances spent an much less time in the upper part of the tank compared with the control group (*P* = 1.6 × 10^−16^, **Figure 1C**), but showed a significantly higher frequency of freezing bouts, relative to controls (*P* = 1.7 × 10^−5^, **Figure 1D**). Additionally, alarm substances exposed zebrafish also spent much more time in immobility (*P* = 0.001, **Figure 1E**) and exhibited much higher frequency in erratic movement (*P* = 8.7 × 10^−6^, **Figure 1F**). Given that the level of cortisol is a good indicator of the degree of stress experienced by fishes (Wendelaar Bonga, 1997), we further examined the cortisol levels, and found that the whole-body cortisol levels were significantly increased when fishes were exposed with alarm substances after 10 minutes compared with control (*P* = 0.009, **Figure 1G**). Taken together, these results indicate that alarm substances can induce dramatically behavioral and physiological changes in zebrafish, similar to the results reported previously (Ogawa et al., 2014; Oliveira et al., 2014).

### Broad-Scale Differences of Gene Expression in Zebrafish After Fright Reaction

In order to disclose the genetic basis underlying the behavior change of zebrafish after exposed to alarm substances, gene expression from olfactory mucosae and brain were characterized using RNA-seq. In total, we sequenced 12 libraries using high-throughput Illumina sequencing producing 150 bp paired-end reads. After quality trimming, more than 21 million reads per trimmed library were mapped to the zebrafish reference genome. On average, approximately 65% of reads were successfully aligned to the assembly across libraries (**Supplementary Table S1**).

**Figure 2:**
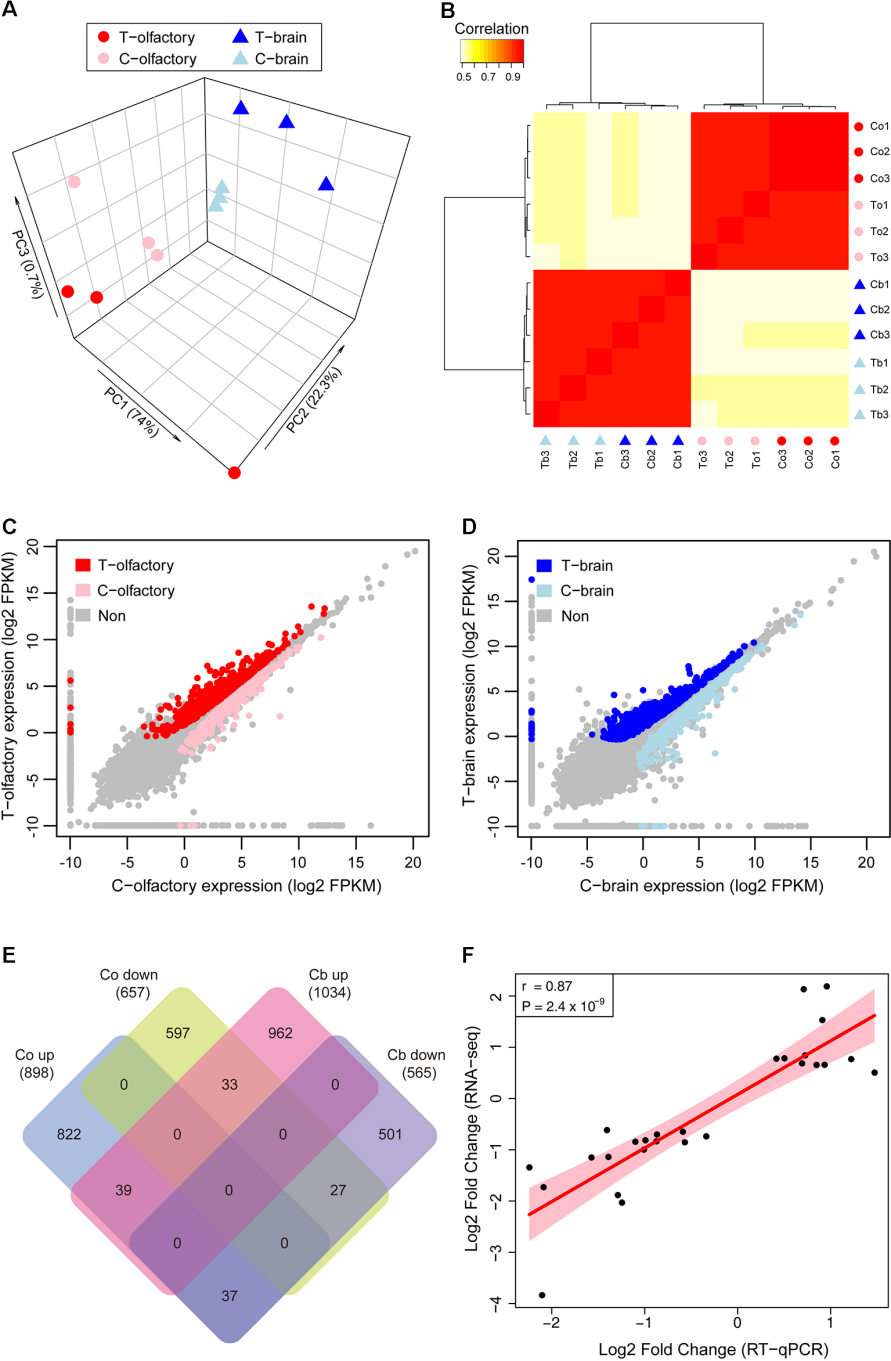
Gene expression patterns of zebrafish after fright reaction. **(A)** Principal component analysis (PCA) plot shows clustering of samples based on treatments and tissues. **(B)** Heatmap of cross correlations of all expression datasets showing different datasets being hierarchically clustered. **(C** and **D)** Pairwise comparisons of gene expression abundances between test group and control group in olfactory mucosae **(C)** and brain **(D)**. **(E)** Venn diagram shows shared genes identified as differentially expressed across tissues. **(F)** RT-qPCR and RNA-seq fold change values are highly correlated. T, Test group; C, Control group.

We first used principal component analysis (PCA) to examine whether there are overall differences of gene expression between treated and untreated samples. PCA results of all expressed genes separated the samples into groups firstly by tissues, and secondarily by experimental treatments (**Figure 2A**), which indicates that tissue and experimental treatment were the major factors explaining variation in gene expression in this study. The hierarchical clustering (HC) analysis further confirmed these results, with the largest divide between tissues and then between experimental treatments (**Figure 2B**). These analyses demonstrated that there had been a considerable shift in gene expression in the olfactory mucosae and brain of zebrafish after exposed to alarm substances.

We next performed differential gene expression analyses to identify genes that significantly contributed to the transcriptome-wide differences between treated and untreated zebrafish across different tissues. In total, we found 1,555 genes that were differentially expressed in olfactory mucosae (**Figure 2C**), among which 898 genes were upregulated and 657 genes were downregulated, respectively (**Supplementary Table S2**). In zebrafish brain, a total of 1,599 genes were identified as differentially expressed genes (**Figure 2D**), with 1,034 genes upregulated and 565 genes downregulated, respectively (**Supplementary Table S2**). Overall, there were more upregulated genes than downregulated genes in both olfactory mucosae and brain, and most of them are differentially expressed in specific tissue (**Figure 2E**).

In order to confirm the expression profiles from RNA-seq data, relative mRNA levels for a total of 27 candidate genes were measured by qPCR. Our results showed that the expression patterns for these genes were highly consistent between RNA-seq and qPCR (r = 0.87, *P* = 2.4 × 10^−9^) (**Figure 2F**, **Supplementary Figure S1** and **Table S3**), suggesting the reliability of our RNA-seq datasets. Interestingly, among these differentially expressed genes, we found that chemokine receptors, including *cxcr3.2* and *cxcr4b*, were significantly uprelugated in olfactory mucosae. Recent study has found that the chemokine-like gene family, *samdori* (*sam*), especially gene *sam2*, plays an important role in fear response in zebrafish (Choi et al., 2018). Another important gene, *v2rl1*, which is a ubiquitously expressed receptor in vertebrate olfactory system (DeMaria et al., 2013; Wakisaka et al., 2017), showed significant downregulation after fright reaction. Although the molecular mechanisms of reduction of mRNA concentration of chemosensory receptors after odorant stimulation remains unclear, large-scale transcriptional profiling of chemosensory neurons have found that odorants induced a decrease in the transcription of genes corresponding to activated odorant receptors (Jiang et al., 2015; von der Weid et al., 2015). Therefore, our data suggested that the vomeronasal receptor genes (*v2rs*) may have important roles in fright reaction in zebrafish.

### GO Enrichment Analysis of Differentially Expressed Genes

Although it is hard to draw firm conclusions about functional differences based on GO analyses alone (Pavlidis et al., 2012), gene enrichment analysis can still be a useful exploratory tool to get interesting patterns (Perry et al., 2014). To explore these large-scale expression changes in transcriptome into a meaningful context, we performed Gene Ontology (GO) enrichment analyses for both upregulated and downregulated genes identified in both olfactory mucosae and brain in zebrafish.

**Figure 3:**
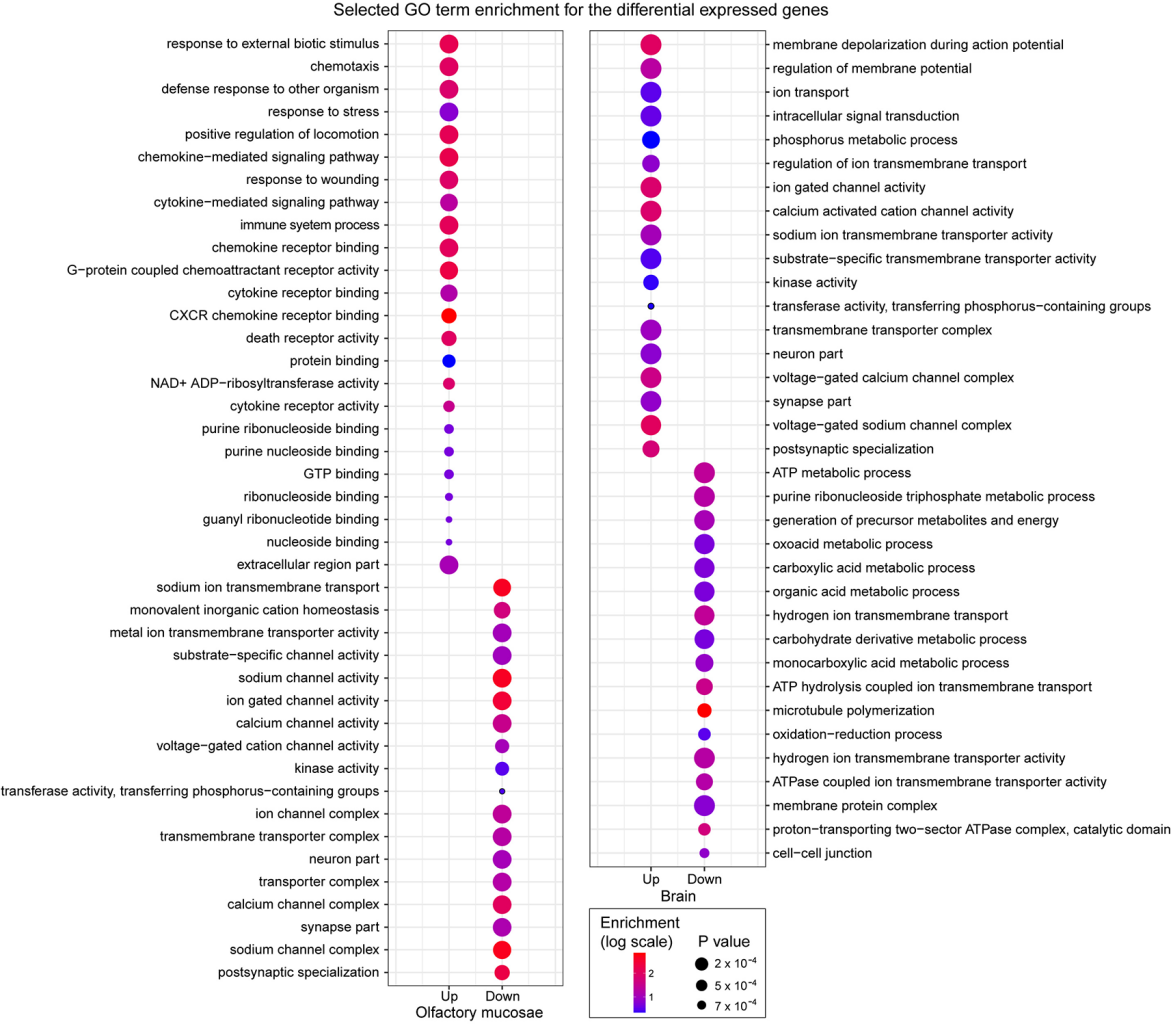
Selected GO term enrichment for the genes identified as differentially expressed in the olfactory mucosae and brain of zebrafish. The enrichment values are indicated in colored scale and p values are indicated with different size.

Interestingly, we observed clear functional differences between upregulated and downregulated genes, and between olfactory mucosae and brain (**Figure 3** and **Supplementary Table S4**). In olfactory mucosae, the upregulated genes were significantly enriched for detecting and responding external stimulus, including response to external biotic stimulus, chemotaxis, positive regulation of locomotion, death receptor activity, guanyl ribonucleotide binding, energy molecular binding, and signal transduction, specifially cytokine mediated signaling pathway. These enriched GO terms indicated that the upregulated genes in olfactory mucosae were directly involved in response to fright reaction in zebrafish. Whereas the downregulated gene in olfactory mucosae was mainly enriched for ion channel activity, including cation homeostasis, sodium channel activity, and calcium channel activity. In brain, the upregulated genes were mainly enriched for signal transduction, including regulation of membrane potential, ion transport, intracellular signal transduction, and neuron part. The dowregulated genes in brain were most enriched for ATP production. In short, these results suggest that differentially expressed genes in zebrafish after fright reaction were indeed associated with their behavior changes.

### Accelerated Evolution for the Fright Reaction Genes

**Figure 4:**
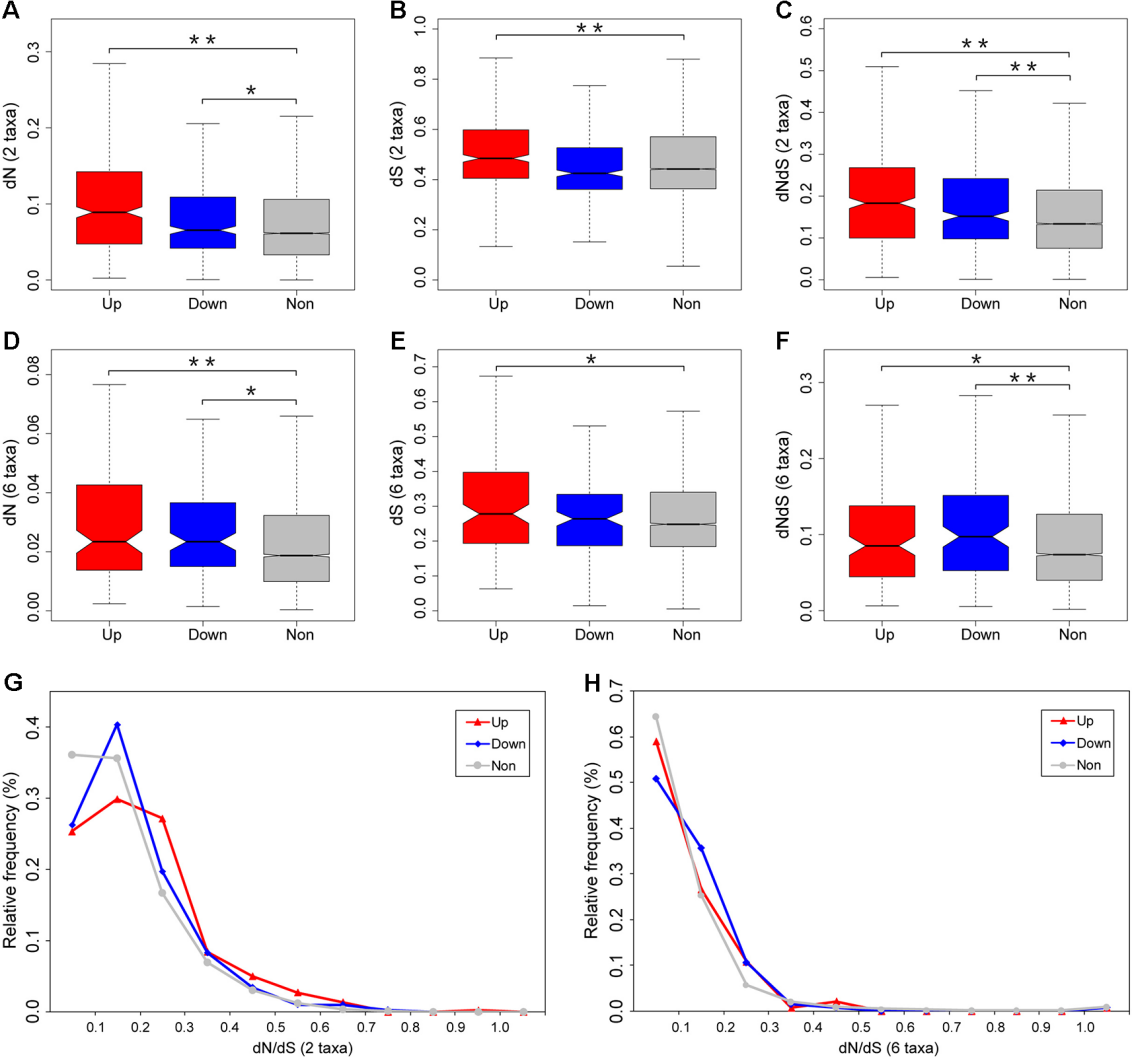
Divergence estimates for the differentially expressed and unbiased genes. Pairwise dN **(A)**, dS **(B)**, and dN/dS **(C)** values were estimated by comparing orthologs between zebrafish and grass carp. Zebrafish lineage-specific dN **(D)**, dS **(E)**, and dN/dS **(F)** values were estimated using six-species alignments among zebrafish, grass carp, cave fish, medaka, stickleback, and spotted gar. The relative frequency distribution of dN/dS ratios for each category of genes were calculated from zebrafish–grass carp comparison **(G)** and zebrafish lineage only **(H)**. Outliers were removed from the boxplot. Significant differences are indicated by the asterisks, based on Wilcoxon rank sum test, *P < 0.05, **P < 0.001.

To determine the impact of fright reaction biased gene expression on rates of gene evolution, we first compared levels of nonsynonymous (dN), synonymous (dS) substitution, and the ratio of nonsynonymous to synonymous substitutions (dN/dS) for differentially expressed and unbiased genes using pairwise comparisons with orthologs between zebrafish and grass carp (**Figure 4**). We found that fright reaction does indeed have a highly significant effect on evolutionary rate of genes. Our results showed that differentially expressed genes (both upregulated and downregulated genes) after fright reaction in zebrafish evolve much faster (i.e., had higher dN/dS ratios) than unbiased genes (*P* = 1.6 × 10^−13^ and *P* = 7.4 × 10^−5^ for upregulated and downregulated genes, respectively. **Figure 4C**). Furthermore, the dN values for the differentially expressed genes were also significantly higher than those for the unbiased genes (*P* = 2.2 × 10^−16^ and *P* = 0.02 for upregulated and downregulated genes, respectively. **Figure 4A**). These results suggested that the higher dN/dS ratios for the differentially expressed genes were mainly come from elevated rates of nonsynonymous substitution (**Figure 4A**), rather than reduced rates of the synonymous substitution (**Figure 4B**). Also, analysis of the frequency distribution of dN/dS ratios showed that the differentially expressed genes tended to be enriched in genes with higher dN/dS ratios and to contain fewer genes under strong selective constraint (dN/dS <0.1) compared with unbiased genes (**Figure 4G**).

Considering that our expression data were taken from zebrafish, it is meaningful to examine the pattern of functional divergence in the zebrafish lineage alone. To do this, we compiled six-species alignments (zebrafish, grass carp, cave fish, medaka, stickleback, and spotted gar) to examine the divergence pattern on the zebrafish lineage since its split from the common ancestor with grass carp. We found that the divergence data for the zebrafish lineage based on the six-species data were qualitatively in accordance with the two-species data, with significantly higher dN/dS ratios for differentially expressed gene than unbiased genes (*P* = 0.04 and *P* = 0.003 for upregulated and downregulated genes, respectively. **Figure 4F**). In addition, the dN ratios were also significantly higher for differentially expressed genes compared with unbiased genes (*P* = 3.6 × 10^−4^ and *P* = 0.001 for upregulated and downregulated genes, respectively. **Figure 4D**). Meanwhile, analysis of the distribution of relative frequency for dN/dS ratios further showed that differentially expressed genes tended to be enriched in genes with higher dN/dS ratios and to contain fewer genes under strong selective constraint (dN/dS <0.1) compared with unbiased genes (**Figure 4H**). Taken together, our results, based on both zebrafish–grass carp pairwise comparison and the zebrafish lineage-specific divergence data, provides convergent evidence that differentially expressed gene after fright reaction exhibit accelerated evolutionary rates in zebrafish.

### Evidence for Positive Selection on Fright Reaction Genes

**Figure 5:**
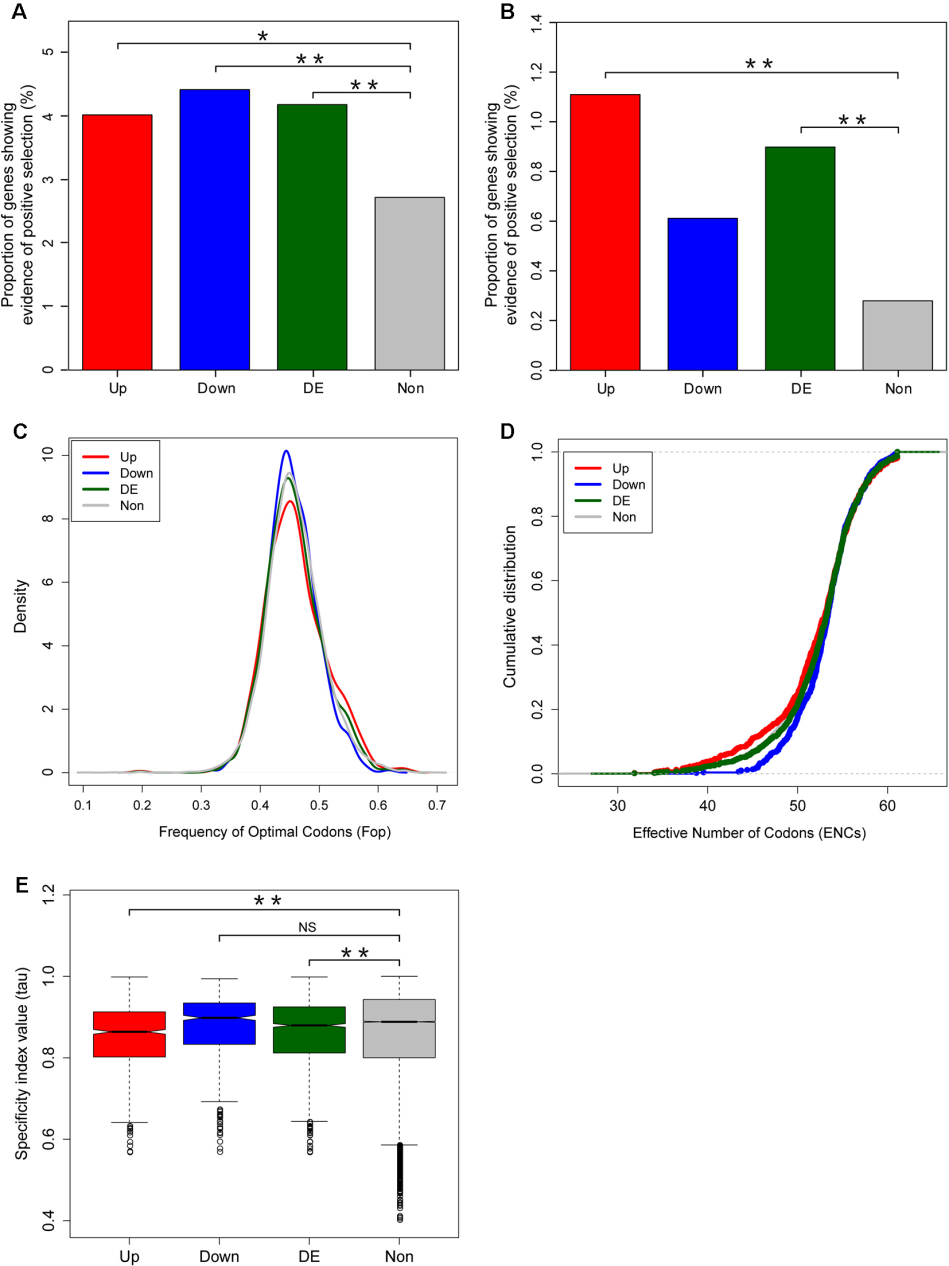
Adaptive evolution of fright reaction genes in zebrafish. Comparison of the proportions of genes showing evidence of positive selection between differentially expressed and unbiased genes. The percentage of positively selected genes was identified using site mode **(A)** and branch site model **(B)** using codeml. **(C**, **D)** Codon usage bias in differentially expressed versus unbiased genes, as measured by frequency of optimal codons (Fop) **(C)** and effective number of codons **(D)**. **(E)** Breadth of expression between differentially expressed and unbiased genes measured by the specificity index value (*τ*). Significant differences were indicated by the asterisks, based on Chi-squared test, *P < 0.05, **P < 0.001. NS means non significant.

In order to assess whether the accelerated evolution of differentially expressed genes was due to increased positive selection or relaxed purifying selection, we used sequence divergence data to examine whether positive selection is more effective on the fright reaction genes. In total, we detected evidence of positive selection for 36 out of the 898 upregulated genes, 29 out of the 657 downregulated genes, and 825 out of the 30,395 unbiased genes, respectively, using the paired nested site models (M1a vs M2a; M7 vs M8) implemented in codeml (PAML4) (Yang, 2007) (**Supplementary Table S5**). Our results indicated that the percentages of genes showing evidence of positive selection for the differentially expressed genes were significantly higher than that for the unbiased genes based on site model in codeml (Chi-squared test, P = 0.019, *P* = 0.008, and *P* = 6 × 10^−4^ for upregulated, downregulated, and differentially expressed genes, respectively. **Figure 5A**).

Considering that the site model in codeml only allow dN/dS ratio to vary among sites but not across lineages, we further used the branch-site models (model = 2, NSsites = 2) to detect genes under positive selection in zebrafish lineage only. By setting the zebrafish as the foreground branch, we compared a selection model that allowed a class of codons on the zebrafish branch to have ω > 1 (Model A2, fix_omega = 0, omega = 1.5) with a neutral model that constrained this additional class of sites to have ω = 1 (Model A1, fix_omega = 1, omega = 1) (**Supplementary Table S6**). The positively selected genes identified from the branch-site models also indicated that the proportion of genes showing evidence of positive selection for the fright reaction genes (differentially expressed genes) was significantly higher than that for unbiased genes (Chi-squared test, *P* = 7.6 × 10^−6^, *P* = 0.11, and *P* = 1.7 × 10^−5^ for upregulated, downregulated, and differentially expressed genes, respectively. **Figure 5B**).

Given that accelerated evolution of genes can also result from relaxed functional constrains, we further assessed the effects of relaxed functional constrains on the evolutionary rates of fright reaction genes. We first determined the codon usage bias and found that there was no significant difference of Fop between the differentially expressed genes and unbiased genes (Wilcoxon rank sum test, *P* = 0.889, *P* = 0.07, and *P* = 0.28 for upregulated, downregulated, and differentially expressed genes, respectively. **Figure 5C**). Similarly, another codon usage bias parameter, the ENCs, also show marginally lower or no difference in differentially expressed genes compared with unbiased genes (Wilcoxon rank sum test, *P* = 0.045, *P* = 0.205, and *P* = 0.514 for upregulated, downregulated, and differentially expressed genes, respectively. **Figure 5D**). As lower ENCs values indicate stronger synonymous codon usage bias, our results suggest that the accelerated evolution of fright reaction genes was not resulted from relaxed functional constrains from codon usage bias.

The breadth of expression of a gene is also an important determinant of its rate of evolution (Duret and Mouchiroud, 2000). Thus, we further assessed the effect of the breadth of expression on the evolutionary rates of genes. We determined the breadth of expression of genes using the specificity index (tau) calculated from gene expression data collected from various tissues and organs (see *Materials and Methods*). Our results showed that fright reaction genes had significantly lower specificity index values compared with unbiased genes (Wilcoxon rank sum test, *P* = 3.1 × 10^−10^, *P* = 0.226, and *P* = 7.6 × 10^−5^ for upregulated, downregulated, and differentially expressed genes, respectively. **Figure 5E**), suggesting that the former have a less tendency to be expressed specifically in particular tissues or organs. Therefore, the accelerated evolution of the fright reaction genes were not under relaxed functional constrains. Collectively, these results demonstrated that the accelerated evolution of fright reaction genes is at least partly driven by adaptive evolution.

## Discussion

The explosive diversification of ostariophysan fishes has attracted numerous attention including morphology, phylogeny, biogeography, and genomics (Fink and Fink, 1981; Saitoh et al., 2003; Nakatani et al., 2011; Chen et al., 2013; Arcila et al., 2017; Chakrabarty et al., 2017; Dai et al., 2018). However, we still know little about the genetic basis of their incredible species richness in freshwaters. Interestingly, early comparative physiology has found that most ostariophysans can emit an alarm substance (schreckstoff) (Frisch, 1938), which is considered an crucial innovation driving the successful radiation of ostariophysan fishes (Helfman et al., 2009). In this study, we combined the first gene expression profiling and evolutionary genomics to report patterns of regulatory and sequence evolution of fright reaction genes in zebrafish, a model species for behavioral studies in ostariophysan fishes (Gerlai, 2003; Miklosi and Andrew, 2006).

Recent studies have observed a strong innate avoidance reaction in zebrafish when exposed to diamines (putrescine and cadaverine) or the scent emanated from decaying or dead zebrafish (Hussain et al., 2013; Oliveira et al., 2014). We here showed that the alarm substance, which is released from epidermal alarm substance cells in living zebrafish (Pfeiffer, 1977), can induce not only a defensive behavior but also a hormonal stress response in zebrafish, as reflected by an increase in whole-body cortisol levels. The levels of plasma cortisol are generally used as a stress indicator in larger fish species (Barton and Iwama, 1991; Wendelaar Bonga, 1997). However, for smaller fisher, such as zebrafish, there is inadequate blood volume to accurately measure plasma cortisol, whole-body cortisol is often employed (Pottinger et al., 2002; Ramsay et al., 2006). Therefore, our data demonstrated that the alarm substances from injured living zebrafish skin can induce remarkable stress reaction on conspecies, both on behaviour, as previously reported (Frisch, 1938; Pfeiffer, 1963), and on physiology.

Our results from transcriptome profiling further show a strong gene expression response to alarm substances. Clear evidence for differential expression was observed in genes involved in response to external stress stimulus, receptor binding, and signal transduction. These data suggests a robust early activation of sensory pathways in olfactory mucosae and brain of zebrafish, as our RNA-seq data was generated from start stage within only about five to ten seconds after fright reaction happened. Thus, our results have important implications for not only understanding the evolution of phenotypic plasticity but also analyzing the variation within transcriptomic data. Although there are still some uncertainties about the real chemical identity of alarm substances that triggers fright reaction (Mori, 2014), several studies have reported two structurally unrelated molecules, hypoxanthine-3-N-oxide (H3NO) (Pfeiffer et al., 1985; Brown et al., 2000) and chondroitin sulfate, as candidates of fish alarm substances (Mathuru et al., 2012). Therefore, a strong gene expression response to alarm substances likely reflects a proximate response to these two components of chemical stimulus.

Vomeronasal receptor families, V1Rs and V2Rs, are suggested to be associated with pheromone detection in vertebrates (Rodriguez et al., 2000), with V1Rs binding to small volatile chemicals (Boschat et al., 2002) and V2Rs binding to water-soluble molecules (Leinders-Zufall et al., 2004; Kimoto et al., 2005). Although some studies suggested that V1Rs are implicated in pheromone detection in fish (Pfister and Rodriguez, 2005; Ahuja and Korsching, 2014; Behrens et al., 2014), none of them were identified as differentially expressed genes in our transcriptome profiling. This is probably due to the fact that the expression levels of V1Rs are generally low, as found in other fishes (Bazaes et al., 2013; Churcher et al., 2015; Cui et al., 2017). However, we did detect a total of 10 out of the 56 V2R receptors (Saraiva et al., 2015), including *v2rl1*, *v2rx3*, *v2rh32*, *v2ra17*, *olfcq19*, *v2rh14* (**Supplementary Table S2**), displaying differential expression after fright reaction. Interestingly, all of these differentially expressed V2R receptors were down-regulated in olfactory mucosa. Though we do not know the reason why *v2r* genes down-regulated after alarm substances stimulus, two recent large-scale transcriptional profiling of chemosensory neurons have found that odorants can activate its receptor genes and induce a fast decrease in the transcription of their receptor genes (Jiang et al., 2015; von der Weid et al., 2015). On the other hand, the gene, *v2rl1*, is a co-receptor expressed with other V2R receptors in all the V2R-positive olfactory sensory neurons (OSNs) in the olfactory epithelium of zebrafish (DeMaria et al., 2013). When the V2R-positive OSNs were activated, the ubiquitously expressed receptor gene, *v2rl1*, would show significant signal. Thus, *v2rl1* is always used as a marker for all the V2R receptors for odorants stimulation (Wakisaka et al., 2017). Together, these observations suggested that vomeronasal receptors type 2 (v2rs) may act as candidate receptors for the alarm substances. However, much more work including pERK immunohistochemistry, Ca^2+^ activity imaging, and neural circuit analyses needs to be done to confirm this hypothesis.

Organisms are able to adaptively regulate gene expression levels in response to environmental changes (Schlichting and Pigliucci, 1998; West-Eberhard, 2003), which is called plastic gene expression. Plastic gene expression have been assumed to be underlie phenotypic plasticity (King and Wilson, 1975), and relative to ubiquitously expressed genes, plastically expressed genes generally evolve under different selection regimes (Schrader et al., 2017). The most notable examples of evolution of plastically expressed genes are from sex-biased genes in fruit flies (Zhang et al., 2004), fishes (Yang et al., 2016; Pauletto et al., 2018), birds (Mank et al., 2007), and mammals (Torgerson et al., 2002), and morph-biased genes in pea aphid (Purandare et al., 2014) and tetraphenic ant (Schrader et al., 2017). All of these studies found that plastically expressed genes tend to evolve faster than uniformly expressed gene. Here, our results shown that the differentially expressed genes after fright reaction also evolve faster than unbiased genes. The similarity in patterns of evolution of differentially expressed genes to that of sex- or morph-biased genes suggests that there may be similar evolutionary patterns for plastically expressed genes, regardless of whether they are short or long term plastic genes.

As any organismal trait, it has been assumed that phenotypic plasticity is always subject to selection and evolutionary change (Schrader et al., 2017), which strengthens the need to assess how plasticity of a trait is affected by selection and whether the plasticity is adaptive (Borenstein et al., 2006). Although it is difficult to disentangle the causes and consequences of accelerated evolution of plastic genes because of possible simultaneous contributions from both adaptive and neutral processes, we still attempted to differentiate these two potential forces by using signatures of selection, codon-bias, and tissue-specificity. Our data firstly showed that the differentially expressed genes have a higher proportion displaying signature of selection than that of unbiased genes based on both site and branch site models. Similar observations have also been found in differentially expressed genes between sexes, such as sex-biased genes in the brown alga *Ectocarpus* (Lipinska et al., 2015), and zebrafish (Yang et al., 2016). Note however that positive selection only impacts a subset of the differentially expressed genes after fright reaction and a significant proportion of them appear to be under relaxed selection. We next evaluated the influence of codon usage bias (Fop and ENCs) on the evolutionary rate of differentially expressed genes and found that there is no difference for the synonymous codon usage bias. The preferential use of a subset of synonymous codons (codon bias) is generally assumed to be driven by functional constrains for favoring efficient and accurate translation (Duret and Mouchiroud, 1999; Duret and Mouchiroud, 2000). Thus, the accelerated evolution of differentially expressed genes after fright reaction was not due to relaxed functional constrains. Finally, we assessed the impact of expression breadth on the evolutionary rates of genes, where broadly expressed genes tend to have more complex functional roles and undergo stronger functional constraints, thus evolve slower than genes with limited expression (Duret and Mouchiroud, 2000). On the contrary, the differentially expressed genes showed a much more expression breadth, implying more stronger functional constrains. Collectively, these results demonstrated that the evolution of differentially expressed genes after fright reaction may be partially driven by positive selection.

## Conclusions

This study represents the first report to investigate the molecular mechanisms underlying the adaptive genome-wide response of zebrafish to fright reaction. Our results demonstrate that the alarm substances have important impacts on zebrafish at multiple levels, including changes in swimming behaviors, cortisol levels, and gene expression patterns. In the future, the identification of the unknown genes detected by our RNA-Seq data should help to further characterize the receptor genes and pathways responsible to the alarm substances.

## Data Availability Statement

RNA-seq data from the sequencing runs were deposited in the Sequence Read Archive (SRA) under accession No. SRP154651.

## Ethics Statement

The animal study was reviewed and approved by the Institutional Animal Care and Use Committee of Institute of Hydrobiology, Chinese Academy of Sciences (Approval ID: Y21304501).

## Author Contributions

SH and TN led this work. LY conceived and designed the projects and performed all computational analyses. LY and HJ performed the experiments. JC, YL, NS, and WL participated in fish sampling. LY and HJ wrote and revised the paper.

## Funding

This work was supported by grants from the National Natural Science Foundation of China (31972866 and 31601858) and Institute of Hydrobiology, Chinese Academy of Sciences (Y55Z09 and Y85E03) to LY, from the National Natural Science Foundation of China (91731301) to SH. The research was supported by the Wuhan Branch, Supercomputing Center, Chinese Academy of Sciences, China

## Conflict of Interest

The authors declare that the research was conducted in the absence of any commercial or financial relationships that could be construed as a potential conflict of interest.

## Supplementary Material

Supplementary Figure S1 Validation of RNA-seq data using qPCR.

Supplementary Movie S1 Alarm responses of zebrafish.
