# Supplementary figures and images for: Comparative Genomics Reveals Accelerated Evolution of Fright Reaction Genes in Ostariophysan Fishes
Source: Front Genet. 2019 Dec 23;10:1283. doi: 10.3389/fgene.2019.01283 (PMC6936194; doi:10.3389/fgene.2019.01283)

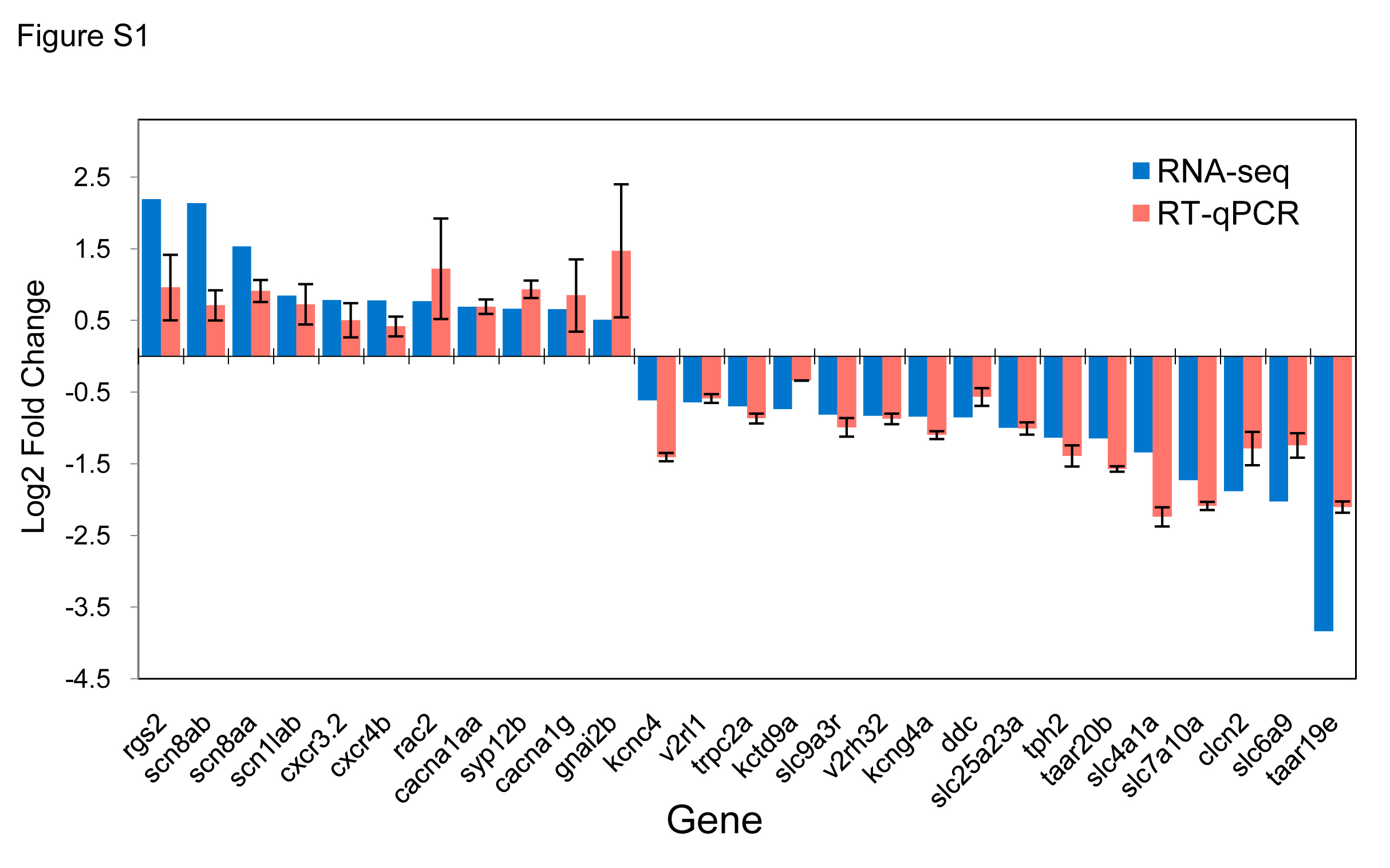

Supplement: Supplementary Figure S1 — Validation of RNA-seq data using qPCR. [file Image_1.jpeg]
